# Integrated single cell-RNA sequencing and Mendelian randomization for ischemic stroke and metabolic syndrome

**DOI:** 10.1016/j.isci.2024.110240

**Published:** 2024-06-11

**Authors:** Jie Li, Sen Shen, Cong Yu, Shuchen Sun, Ping Zheng

**Affiliations:** 1 Department of Neurosurgery, Shanghai Tongren Hospital, Shanghai Jiaotong University School of Medicine, Shanghai, China; 2 Department of Neurosurgery, Shanghai Pudong New area People’s Hospital, Shanghai, China

**Keywords:** Health sciences, Medicine, Medical specialty, Internal medicine, Cardiovascular medicine, Omics, Transcriptomics

## Abstract

Although more and more evidence has supported that metabolic syndrome (MS) is linked to ischemic stroke (IS), the molecular mechanism and genetic association between them has not been investigated. Here, we combined the existing single-cell RNA sequencing (scRNA-seq) data and mendelian randomization (MR) for stroke to understand the role of dysregulated metabolism in stroke. The shared hub genes were identified with machine learning and WGCNA. A total of six upregulated DEGs and five downregulated genes were selected for subsequent analyses. Nine genes were finally identified with random forest, Lasso regression, and XGBoost method as a potential diagnostic model. scRNA-seq also show the abnormal glycolysis level in most cell clusters in stroke and associated with the expression level of hub genes. The genetic relationship between IS and MS was verified with MR analysis. Our study reveals the common molecular profile and genetic association between ischemic stroke and metabolic syndrome.

## Highlights

• The diagnostic model of metabolic-related genes can distinguish the ischemic stroke
• There is similar immune infiltration between ischemic stroke and metabolic syndrome
• Shared metabolic pathways are found between ischemic stroke and metabolic syndrome

## Graphical abstract

**Figure:**
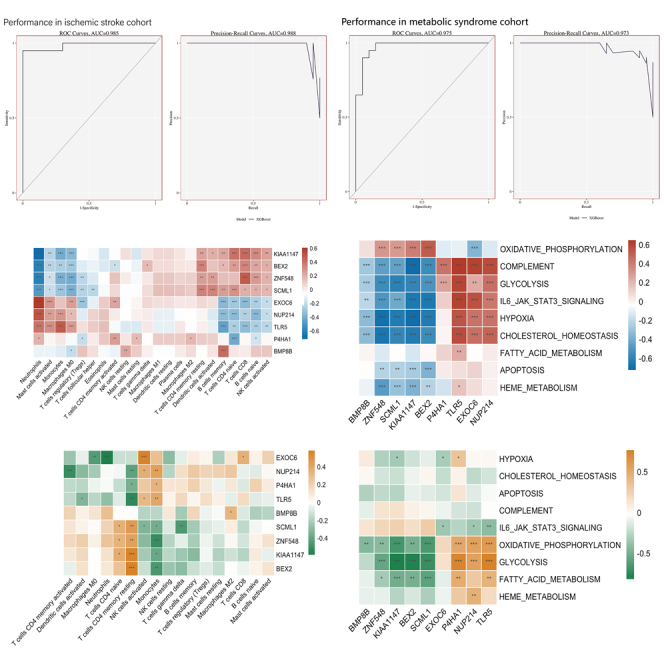

## Introduction

Ischemic stroke (IS) is a common neurological disease and its onset is about 7.6 million every year.^1^ The clinical treatment of IS is surgical interventions together with medicines.^2^^,^^3^ However, the exact etiology of IS is unknown, although several risk factors have been identified for decades including the metabolic syndrome (MS). The MS is associated with increased risk of stroke (Hazard ratio = 1.5), and the reduction of MS would lead to a 19% reduction in stroke for both women and men.^4^ Around 50–60% stroke patients are found to have metabolic dysregulation.^5^ The pharmacological targeting of MS includes lifestyle optimization and drugs using. However, the direct link between stroke and MS is missing, especially regarding the genetic risks, which is promising and has attracted more attention in clinical studies.

A series of studies indicate MS can increase the rate of stroke. In one study, patients with the MS without diabetes exhibited a 1.49-fold increased odds ratio (OR) for IS; while, MS patients with diabetes had a 2.29-fold increased OR for IS.^6^ The definition of MS is a little bit different based on different populations including several items: waist circumference, blood pressure, fasting plasma glucose, serum triglycerides and high-density lipoprotein cholesterol. We recently proposed a risk model for clinical outcome prediction in acute stroke patients with intravenous thrombolysis and found identified both hypertension and dyslipidemia are risk factors of poor neurological outcome.^7^ Although several studies have identified the metabolism-related syndrome in stroke, and MS is considered as an independent risk factor for IS with poor neurological outcome.^8^ Interestingly, a recent study from Jae-Young Lee group found stroke is also a risk factor for MS with an odds ratio at 2.003.^9^ These findings indicate that stroke and MS can impact each other. However, metabolism-related genes in stroke is not clear, which needs to be further investigated.

Therefore, in our study, we aimed to explore the association between stroke and metabolism using integrated bioinformatics methods, and weighted gene co-expression network analysis (WGCNA) was then used to identify the key module associated with both diseases. Candidate hub genes were then identified within the key modules. Based on the sc-RNA seq, cellular hub genes were identified with cell-cell interactions. Potential clinical significance of these genes was then determined by machine learning models and further validated with the receiver operating characteristic curve analysis. We hope that this research can offer new insights into significant diagnostic biomarker and potential therapeutic targets for treating stroke.

## Results

### The DEGs in both ischemic stroke and metabolic syndrome

**Figure 1:**
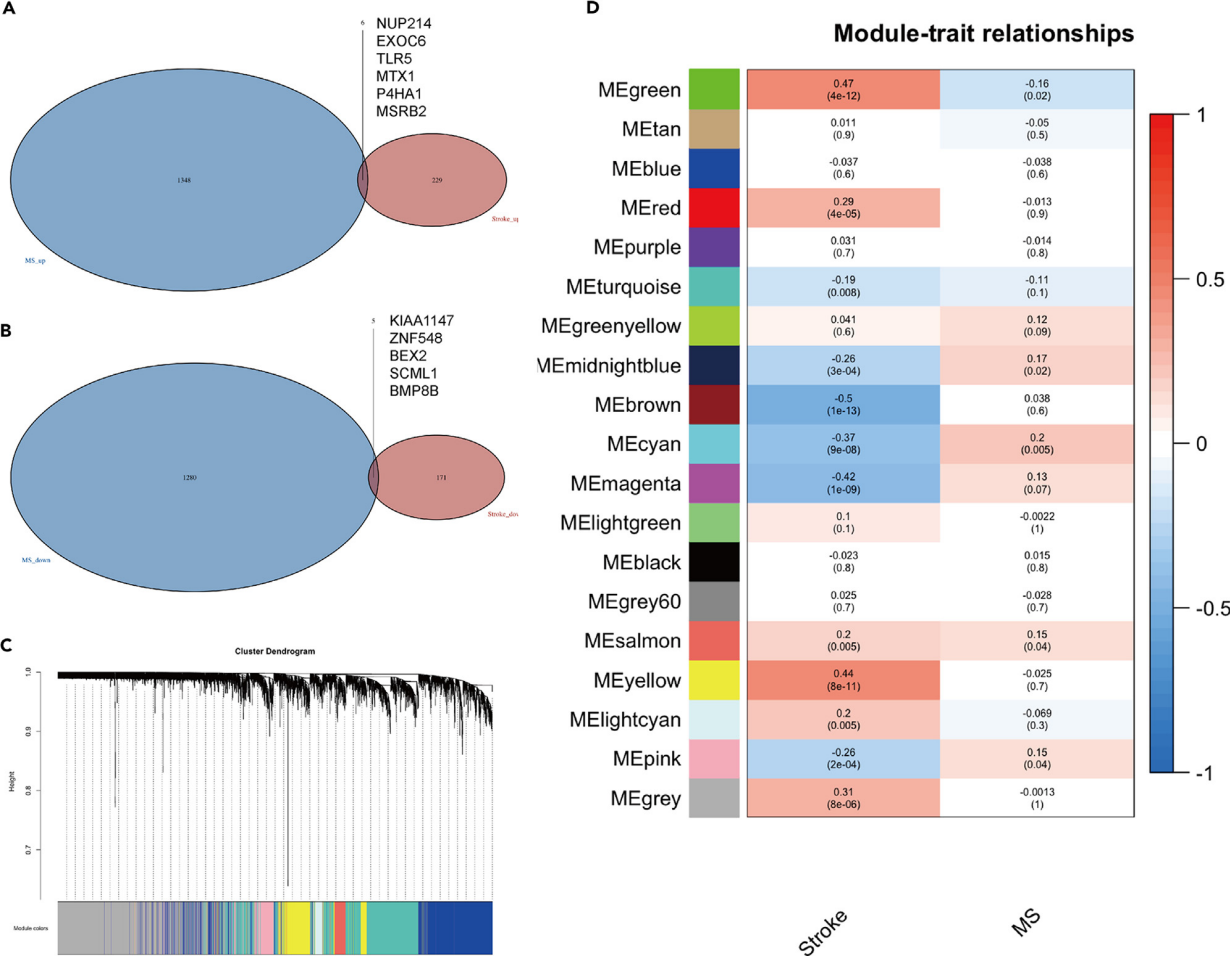
The shared hub genes in ischemic stroke and metabolic syndrome (A) The Venn figure of six upregulated DEGs in both stroke and MS. (B) The Venn figure of five downregulated DEGs in both stroke and MS. (C) WGCNA analysis shows the modules related to both stroke and MS. (D) Module-trait relationships for the stroke-based WGCNA and MS-based WGCNA.

We investigated the differential genes in stroke and metabolism syndrome, firstly, we combined the stroke datasets together with MS dataset (Figure S1) and we found 1348 upregulated and 1280 downregulated genes in stroke (Figure 1A); while there were 229 upregulated genes and 171 downregulated genes in MS (Figure 1B). To identify the association between stroke and MS, we crossed these genes and obtained six co-increased genes and five co-decreased genes in both diseases. In addition, we combined these genes and applied WGCNA to link these genes with clinical characteristics (Figure 1C). We obtained 19 modules (one gray module), according to the module-trait relation figure, we found the MEsalmon module has a positive relationship with both stroke (r = 0.2, *p* = 0.005) and MS (r = 0.15, *p* = 0.04), and this indicated that these genes might be involved in both diseases.

#### The machine learning models based on DEGs

**Figure 2:**
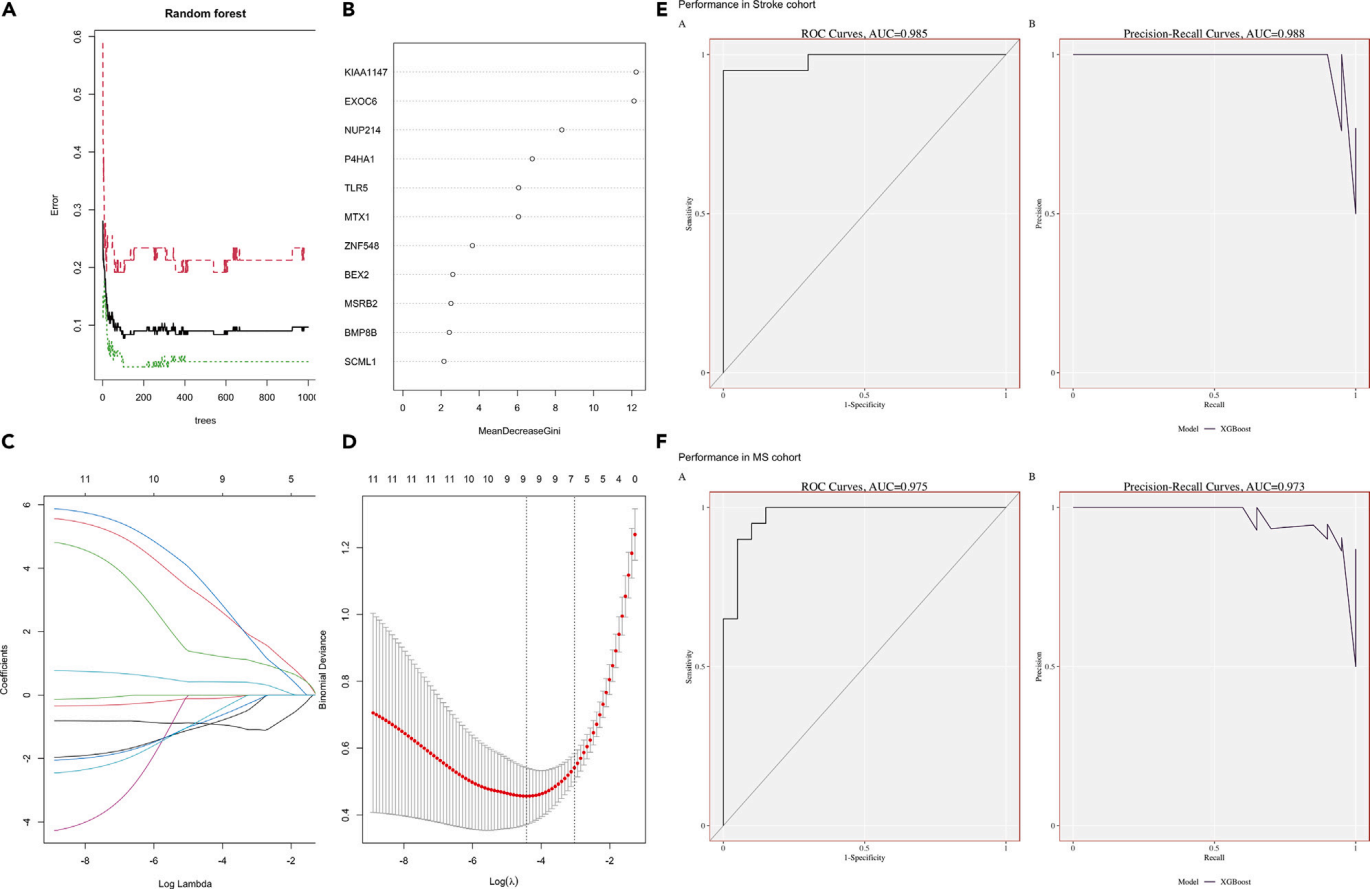
The diagnostic model for both stroke and MS (A) The importance plot of random forest show the prediction of patients with stroke (red lines); patients without stroke (green lines) and the potential prediction error (black lines). (B) The gene rank in random forest based on mean decrease Gini value. (C and D) Lasso regression to select 9 hub genes for the regression model. (E) The ROC and PRC validation of the diagnostic model in external stroke dataset. (F) The ROC and PRC validation of the diagnostic model in MS dataset.

To construct the diagnostic model, we applied the randomForest method in these eleven co-expressed genes with the “randomForest” package, and obtained the importance plot and the gene rank based on the mean decreased Gini value (Figures 2A and 2B). To further eliminate the co-linear characteristics, we used the Lasso method with “glmnet” package and obtained nine genes for the Lasso regression (Figures 2C and 2D). The Lasso equation is (−0.908∗KIAA1147 + 2.969∗EXOC6+1.281∗NUP214 + 3.378∗P4HA1+0.420∗TLR5-0.936∗ZNF548–0.111∗BEX2-0.805∗BMP8B-0.671∗SCML1). To validate the diagnostic efficiency of this model, we first applied the XGBoost and validated this model in the external stroke data and metabolic dataset. After the cross-validation and Bayes optimization with 20 rounds, we selected the best super-parameter for both XGB models (Figure S2) and plotted ROC for the nine hub genes with AUC>0.7 in stroke dataset (Figure S3). The ROC and precision recall-curves of the diagnostic model was also validated in the external stroke model and MS with an AUC at 0.985 and 0.975, respectively; while the PRC value is 0.988 and 0.973, respectively.

### The immune infiltration and metabolic pathways of hub genes

**Figure 3:**
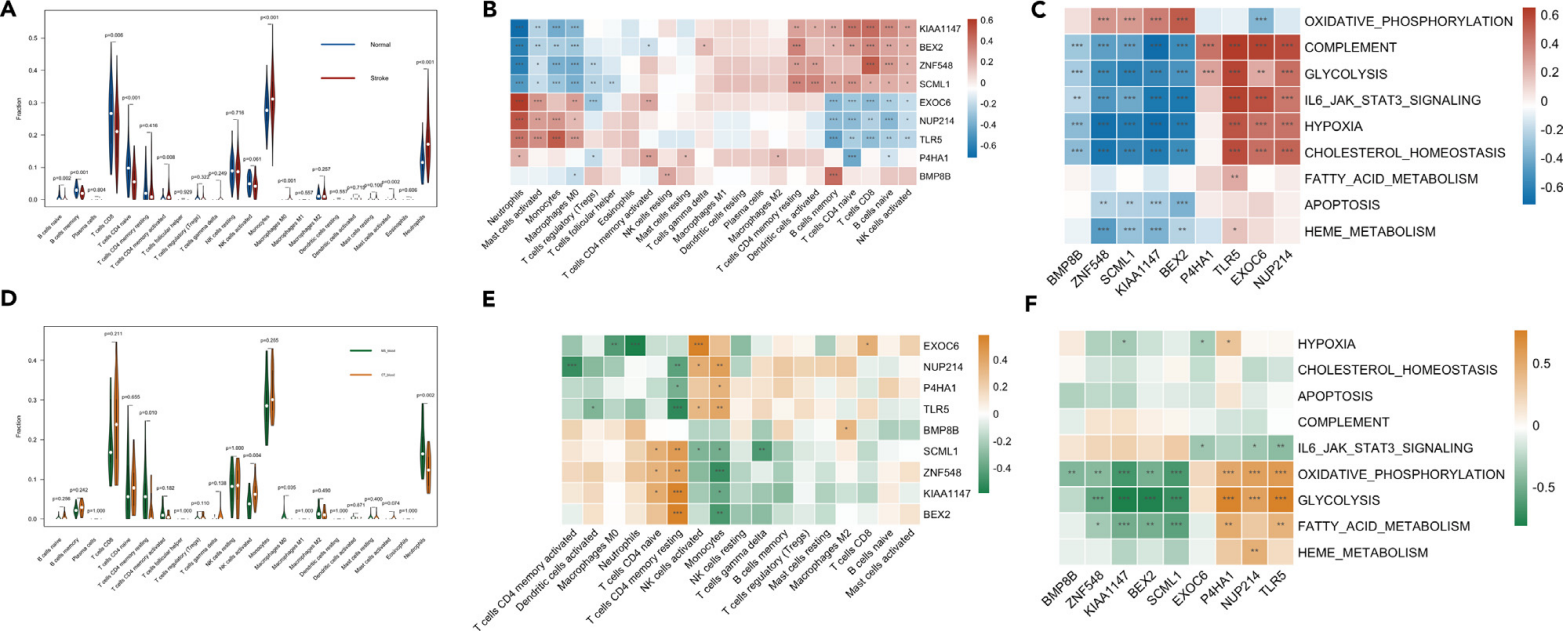
The comparison of immune infiltration and metabolic pathways in stroke and MS (A and D) Boxplot shows the immune infiltration between the two groups. (B and E) The heatmap shows the correlation between hub genes and infiltrated immune, inflammatory cells between the two groups. (C and F) The heatmap shows the correlation between hub genes and metabolic pathways between the two groups.

To further elaborate the shared pathways between IS and MS, we further compared the immune infiltration and metabolic pathways between the two groups and found the stroke group has a lower infiltration of B cells (naive, memory), T cells (CD8, CD4 naive) and enrichment of neutrophils, M0 macrophages and monocytes suggesting that IS group has immune deficiency and inflammation phenotype. Meanwhile, the MS group has a deficiency in resting CD4 memory T cells, and enrichment in neutrophils as well (Figure 3).

In addition, we investigated the relationship between risk genes and infiltrated immune cells. We found Toll-like receptor 5 (TLR5) is positively correlated with infiltrated monocytes in both diseases; Prolyl 4-hydroxylase subunit alpha-1 (P4HA1) is also positively associated with the neutrophils in stroke and monocytes in MS; while it is negatively associated with naive CD4 T cells and Tregs in stroke; and negatively associated with resting memory CD4 T cell in MS. Interestingly, brain expressed X-linked 2(BEX2) is positively correlated with resting memory CD4 T cell and negatively with monocytes in both diseases. We further investigate the correlation between risk genes and metabolic pathways with GSVA packages. We found several shared pathways in these two diseases and dominated by the same genes. TLR5 and P4HA1 are positively associated with glycolysis in both diseases; while BEX2 is negatively associated with glycolysis in both stroke and MS.

### Single cell RNA sequencing for metabolic-related genes in ischemic stroke

**Figure 4:**
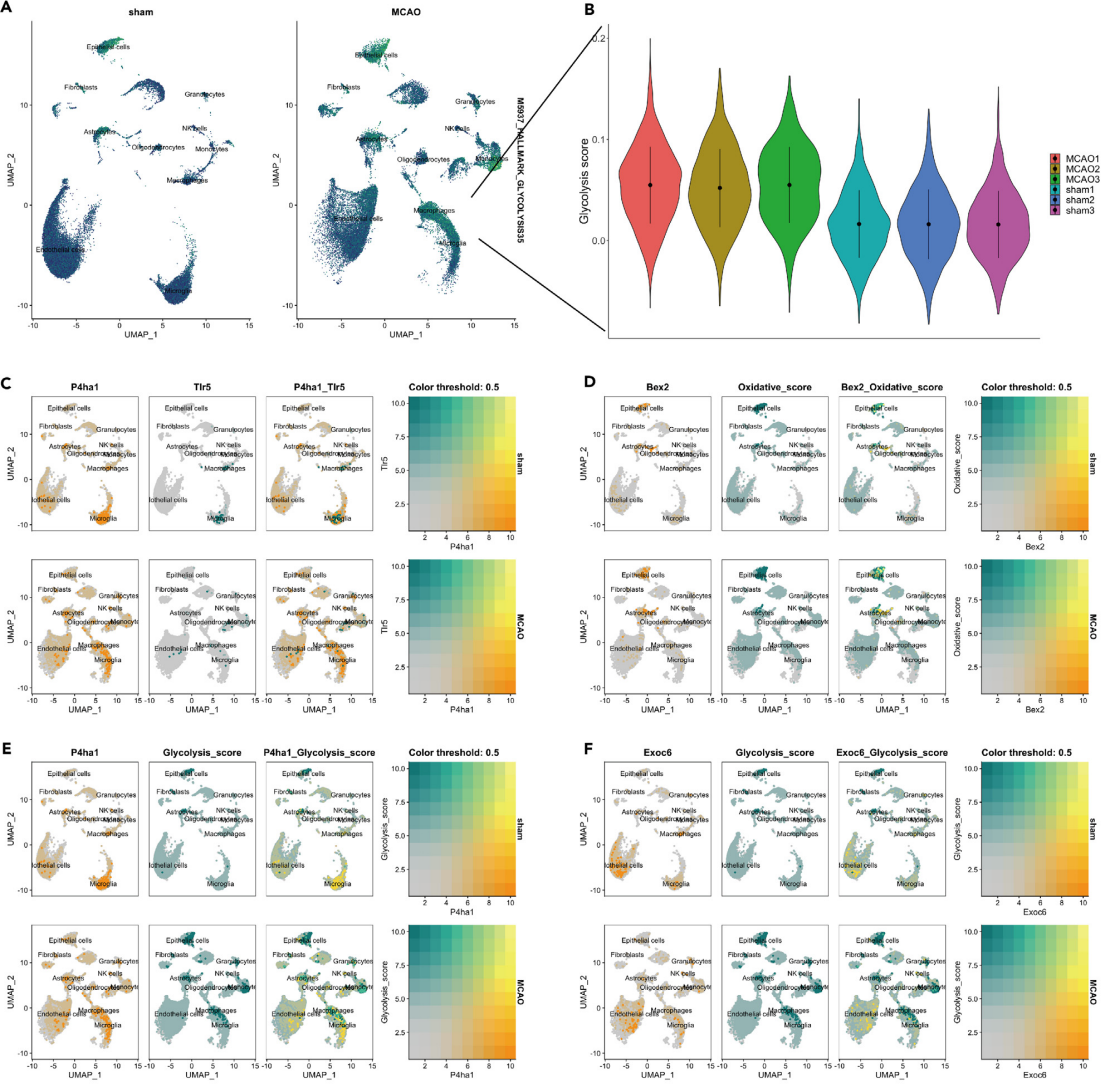
The glycolysis score comparison between MCAO mouse and sham mouse (A) The glycolysis score between the two groups assessed by addmodulescore. (B) Violin plot shows the quantitative comparison of glycolysis score in microglia between MCAO and sham group. (C) The co-expression of P4ha1 and Tlr5 on the uMAP. (D) The co-expression of Bax2 and oxidative score on the uMAP. (E) The co-expression of P4ha1 and glycolysis score on the uMAP. (F) The co-expression of Exoc6 and glycolysis score on the uMAP.

To further clarify the role of metabolic-related genes in IS, we analyzed the sc-RNA seq data from GSE174574, which had three IS and three sham mouse as there was no human stroke sc-RNA seq data available at the moment. The detailed information regarding the samples were reported previously.^10^ We first compared the glycolysis extent in the brains from sham and MCAO mouse in different cell groups (Figures 4A and 4B). The glycolysis level was statistically different in the microglia between MCAO and sham group (*p* < 0.05). As the microglia hold the smallest *p*-value, we took them for reclustering, which showed the obvious difference between sham and MCAO groups (Figure 4A). We mapped the six risk genes in the UMAP (Figure S4). Moreover, we could also identify the microglia glycolysis level was statistically increased in MCAO group compared to the sham group (*p* < 0.05, Figure 4B). We further located the risk gene expression with oxidative and glycolysis score. We found both P4ha1 and Tlr5 were mostly enriched in the microglia; while Bex2 was associated with oxidative status in both astrocytes and epithelial cells; while P4ha1 and Exoc6 were closely associated with the glycolysis score in microglia (Figures 4E and 4F).

**Figure 5:**
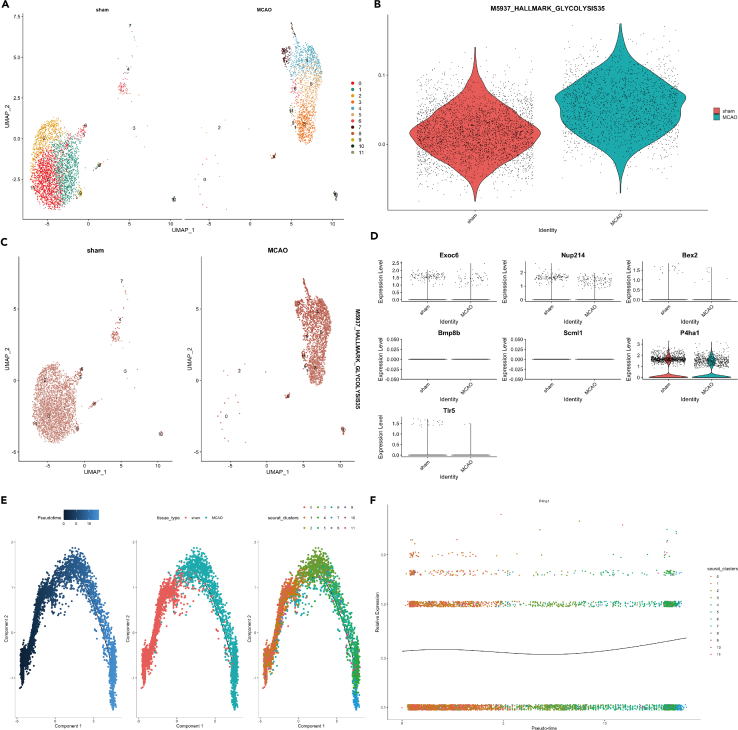
The glycolysis score comparison in microglia between MCAO mouse and sham mouse (A) The sub-cluster for microglia between the two groups. (B) Violin plot shows the quantitative comparison of glycolysis score in microglia between MCAO and sham group. (C) The glycolysis score distribution in microglial subclusters between the two groups. (D) The expression level of hub genes in microglia between sham and stroke. (E) The pseudo-time analysis of the microglia sub-cluster. (F) The pseudo-time analysis shows the hub gene P4ha1 in the stage transition in microglia.

As we found the different microglia clusters between sham and middle cerebral artery occlusion (MCAO), we first reclustered the microglia between sham and MCAO and obtained 11 sub-clusters (Figure 5A). The glycolysis level (M5937) was higher in the microglia of MCAO group compared to the sham group (Figures 5B and 5C). We then applied the pseudo-time analysis to identify the transition of microglia sub-clusters, and we could see a clear stage change of microglia from sham to MCAO and according alteration of P4ha1 gene expression during the transition (Figures 5E and 5F).

**Figure 6:**
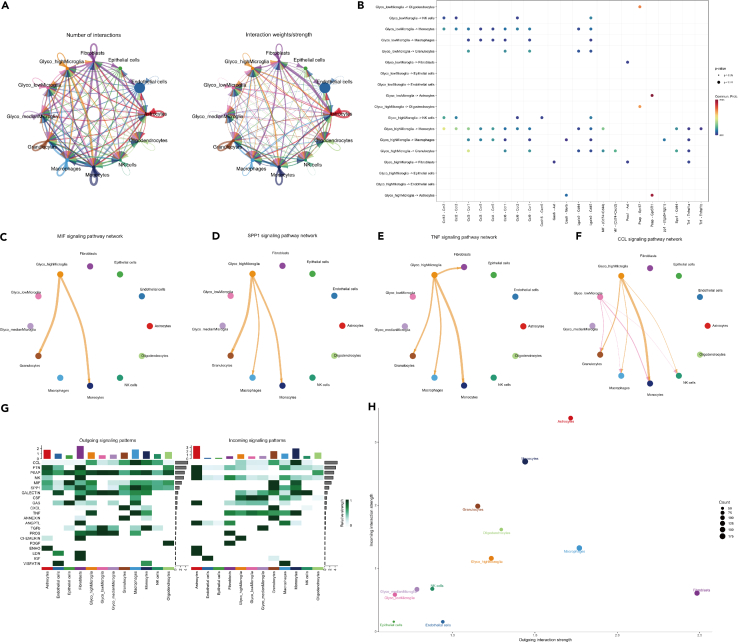
Cell-cell interaction at single-cell level in MCAO (A) The number of interactions and interaction weights between several cell types. (B) The bubble plot shows the relationship between cell types and ligand-receptor. (C–F) MIF, SPP1, TNF and CCL signaling pathway network between several cell types. (G) The outgoing and incoming signaling patterns in different cell clusters with related signaling pathways. The relative strength is shown from shallow to deep color. (H) The correlation map of different cell clusters. The size of the circle indicates the counts of related genes involved. The x axis is outgoing interaction strength and the y axis is incoming interaction strength.

As metabolism-related genes have a critical role in immune infiltration assessed by CIBERSORT, we further explored the cell-cell interaction at a single cell level with CellChat R package. We first found the tight interaction between several cell types (Figure 6A). The bubble plot of the network showed that microglia with high glycolysis has a connection with NK cells via cxcl16/cxcr6, with macrophages and astrocytes via Gas6/Mertk and PP1/Itga5+Itgb1, with monocytes and granulocytes via MIF/CD74+CD44, and fibroblasts via Tnf-Tnfrsf1a/Tnfrsf1b, while this connection missed in microglia with low glycolysis level. These connections suggest that microglial glycolysis has a tight relationship with the inflammatory chemokines and cell signaling (Figure 6B). We then specifically showed the MIF, SPP1, TNF and CCL signaling pathway network among low and high glycolysis microglia, with other cell types (Figures 6C–6F). In addition, we showed the interaction between microglia with high or low glycolysis and other cell types in both outgoing and incoming signaling patterns (Figure 6G). Meanwhile, the microglia with high glycolysis has a more connection with other cells compared to microglia with low glycolysis (Figure 6H).

### MR analysis between ischemic stroke and metabolic disorder

The results of the two-sample MR estimates for IS and metabolic disorder are summarized in Figure S6. We found IS is positively associated with metabolic disorder (OR = 1.36, *p* = 4.85E-06). For the bidirectional study, we found metabolic disorder is also positively correlated with IS (OR = 1.21, *p* = 4.64e-03), which affects each other, and this might explain why they shared the similar gene pathways. The detailed results for the MR between IS and metabolic disorder are in Figure S6.

## Discussions

IS results in a higher rate of morbidity and mortality, which is a common neurological disease with poor prognosis.^11^ We recently constructed a nomogram based on the baseline NIHSS, blood sugar, and blood cholesterol level to predict the neurological outcome in stroke patients with intravenous thrombolysis. Therefore, we focused on the glycolysis pathways in both IS and MS. Previous studies indicate that inflammation, immunity, and metabolic alterations are involved in the occurrence and development of IS.^10^ Neutrophil infiltration is a key marker of metabolic dysregulation in stroke, and we previously showed that suppression of NLRP3 can attenuate hemorrhagic transformation in thromboembolic stroke rats via reducing the neutrophil recruitment.^12^ Regarding the immune cell infiltration, we found that higher neutrophil to lymphocyte ratio (NLR) is associated with hemorrhagic transformation in stroke patients.^13^^,^^14^ However, we did not further investigate the relationship between neutrophil-dominant inflammation phenotype and metabolism markers. This time, we supplemented the sc-RNA seq method to demonstrate the high infiltration of neutrophils and lower level of B cells, T cells in MCAO mouse, especially in microglia with high glycolysis level. We further showed the microglia with high glycolysis extent had increased connection with other cells via different ligand-receptors.

Anaerobic glycolysis is a pathology in stroke; a recent study found one compound, a novel glycogen phosphorylase inhibitor, can reduce anaerobic glycolysis and inhibit the apoptosis in a cerebral ischemic-reperfusion model.^15^ Another compound named dichloroacetic acid (DCA) has also been found to regulate glycolysis and protect the neurons from oxidative damage in I/R injury^16^

Previous study has confirmed that exceeded anaerobic glycolysis in cerebral ischemia can trigger the excess production of lactate and pyruvate, which in turn causes neurodegeneration.^17^ However, whether the glycolysis level is altered in the cellular level in stroke remains unknown. Therefore, to clarify the glycolysis-related genes in IS, we found the glycolysis level varied in different cells and correlated with different genes. First, we found several metabolic pathways shared in two diseases and BEX2 is negatively associated with glycolysis in both stroke and MS, while TLR5 and P4HA1 are positively associated with glycolysis in stroke and MS. In addition, we found these risk genes are also associated with the immune microenvironment. TLR5 is positively correlated with infiltrated monocytes in both diseases; P4HA1 is also positively associated with the neutrophils in stroke and monocytes in MS, while it is negatively associated with naive CD4 T cells and Tregs in stroke and negatively associated with resting memory CD4 T cell in MS. Interestingly, BEX2 is positively correlated with resting memory CD4 T cell and negatively with monocytes in both diseases. These results indicate that BEX2, TLR5 and P4HA1 might be involved in the immune deficiency, inflammation phenotype and abnormal glycolysis in stroke. We showed the expression level of the hub genes at the single-cell level and found Bex2 and Exoc6 were mostly co-expressed in epithelial cells and astrocytes; while P4ha1 and Tlr5 were dominantly co-expressed in macrophages and microglia (Figure S5). The expression pattern of these hub genes was similar between the bulk RNA sequencing and single-cell RNA sequencing data. P4HA1 has recently been found to be a prognostic biomarker in pan-cancer, especially in lung adenocarcinoma.^18^ It is also negatively associated with the CD8^+^ T cells, NK cells; while positively correlated with tumor-associated macrophages, carcinoma-associated fibroblasts and Tregs. P4HA1 is also associated with the glucose metabolism in pancreatic cancer cells (PDAC), where it can enhance the Warburg effect and tumor growth in PDAC.^19^ P4HA1 is also a novel glycolysis-related gene in predicting the survival patients with colon adenocarcinoma.^20^ TLR5 is originally found to increase in rat brains of focal ischemia models, and luteolin can downregulates TLR5-NFκB via reducing the expression of TLR5.^21^ A recent study also reported that TLR5-NADPH oxidase 4 (Nox4) promotes the migration of smooth muscle cells in atherosclerosis and facilitate the formation of atherosclerotic plaque.^22^ Regarding the association between TLR5 and metabolic pathways, TLR5 is shown to activate the glycolysis in myeloid dendritic cells (mDCs).^23^ However, whether this activation mode is also involved in stroke needs to be verified in future studies. BEX2 is a brain-expressed X-linked protein-2, which is widely expressed in different tissues. Its level is highest in brain, testis and the olfactory system.^24^ BEX2 overexpression can protect the breast cancer against mitochondrial apoptosis, via the modulation of Bcl-2 protein family and BAD, BAK1.^25^ The same group recently reported that BEX2 also promotes the proliferation of glioma cells via JNK pathways and NF-κB p65.^26^ The similar pathway of BEX2-JNK has also been reported in colorectal cancer to promote the tumor proliferation.^27^ These results indicate that BEX2 has an oncogenic effect in pan-cancer, and promotes the cell proliferation, which might become a target in stroke to protect the neurons from pan-apoptosis.

As the microglia has the biggest difference in glycolysis score, we took them for further sub-cluster analysis. We found two stages of microglia from sham- to stroke-based on the pseudo-time analysis and the risk gene: P4ha1 has a critical role in the stage transition. When we classified the microglia with high and low glycolysis, we found only microglia with high glycolysis level had a tight cell-cell interaction with monocytes, macrophages, granulocytes, and fibroblasts via MIF, SPP1, TNF, CCL, and other ligand-receptors, while this connection missed in microglia with low glycolysis level. Knockout studies in mice also suggest a role of this gene in protecting neurons from apoptosis by stimulating antioxidative pathways and inhibiting inflammatory pathways.^28^ Tumor necrosis factor-α (TNFα) is a specific pro-inflammatory cytokine, which is highly expressed in stroke. It can bind to two receptors: TNF receptor superfamily member 1A (TNFRSF1A) and TNF receptor superfamily member 1B (TNFRSF1B). Depletion of Tnfrsf1a is able to form the neuroblasts and subventricular zone cell proliferation.^28^ Administration of a soluble TNF inhibitor can reduce the infarct volume in MCAO mice.^29^ A current study reported the different role of TNFR1 and TNFR2 in demyelination disease, which is similar to M1 and M2 roles.^30^ However, these studies did not elucidate the source of TNF. In our study, we applied the cell-chat analysis to find microglia secret MIF, SPP1, TNF, and CCL to monocytes and granulocytes, macrophages. This is the first time that the microglial glycolysis is linked to TNF and CCL in stroke. Furthermore, TNFα is thought to be a triggering factor in thrombus via activation of complement system.^31^ The complex relationship between glucose metabolism and coagulation in stroke is well deserved to be investigated in future studies.

Liang et al. found that MS is positively associated with the likelihood of coronary heart diseases in stroke patients with an OR at 1.27, which indicated that metabolism dysregulation not only impacts the brain vessels, and it also had a critical effect in heart vessels.^32^ In addition, Adeoye et al. also reported that stroke patients with MS have abnormal blood triglyceride and fasting glucose level, stroke severity and fatality complications.^33^ In addition, some inherited neurometabolic disorders also demonstrate the stroke-like episodes.^34^ A large study of 114 out of 3904 participants with atherosclerosis concluded that four metabolites: methionine sulfoxide, N6-acetyllysine, sucrose and glucuronate were associated with increased risk of stroke. The constructed a stroke metabolite score (SMS) based on the expression of these four metabolites and found it has a prediction ability of AUC at 0.65. However, our risk models based on the metabolism-related genes can improve the prediction ability to more than 97–98%,^35^ as these hub genes are obtained from the DEGs in both diseases. A recent review paper has indicated that gut bacterial dysbiosis is closely associated with the hypertension, diabetes, obesity, dyslipidemia, and MS, which are the main risk factor for stroke, and the authors also proposed that the gut bacterial-derived metabolites might be involved in the IS.^36^ Hydrogen can protect mice from IS via modulating several endogenous metabolites enriched in glutathione, taurine pathway and mitochondrial energy metabolism.^37^ However, these studies did not further elucidate the role of metabolism-related genes in stroke.

### Conclusion

In conclusion, our research combined the analysis results of bulk RNA-seq and scRNA-seq data revealing metabolism-related hub genes and their role in immune and inflammation phenotype in stroke, which can provide useful biomarkers and interventional strategy.

### Limitations of the study

There are also several limitations that need to be addressed in our study. First, the data we used was from public databases, which were limited in the sample size. Further research with larger sample sizes from multi-centers are essential to validate our results. Second, the functions and potential molecular mechanisms of genes are quite complicated and further experimental verification of cellular and animal is required. In this study, nine hub genes associated with the probability of both IS and MS were identified through the GEO databases. Then, a diagnostic model was established and a risk score was constructed for prediction and verification. However, the exact role of these metabolism-related genes in stroke needs to be validated in further experiments.

## STAR★Methods

### Key resources table

**Table:** 

REAGENT or RESOURCE	SOURCE	IDENTIFIER
**Deposited data**		

https://www.ncbi.nlm.nih.gov/geo/query/acc.cgi	GSE58294	GEO dataset
https://www.ncbi.nlm.nih.gov/geo/query/acc.cgi	GSE16561	GEO dataset
https://www.ncbi.nlm.nih.gov/geo/query/acc.cgi	GEO174574	GEO dataset

**Software and algorithms**		

Seurat versions 4.1.3	Zhang et al.,^38^ 2021; Johanses et al., 2023	https://satijalab.org/seurat/; RRID: SCR_016341

### Resource availability

#### Lead contact

Further information and requests for resources and reagents should be directed to and will be fulfilled by the Lead Contact, PZ (zhengping@shpdph.com).

#### Materials availability

This study did not generate new unique reagents.

#### Data and code availability

• The data are from the public dataset and listed in the STAR methods.
• This paper does not report original code. Any additional information required to reanalyze the data reported in this work paper is available from the lead contact upon request.

### Experimental model and study participant details

No human or animal experiments were involved in this study, and the ethics were not warranted.

#### Data download

We downloaded the datasets (GSE58294, GSE16561) from NCBI website.^39^^,^^40^ GSE58294 (GPL570) has ninety-two blood samples including 69 ischemic stroke patients and 23 healthy controls. The GSE16561 dataset (GPL6883) has 39 ischemic stroke patients, and 24 healthy controls. GSE22255 has 20 ischemic stroke patients and 20 healthy controls was used as the external validated datasets. We also included the GSE98895 dataset,^41^ which had 20 patients with metabolic syndrome and 20 healthy controls.

#### Data preprocessing and metabolism-related differentially expressed gene screening

The Microarray data processing, including normalization and correction with the Affy and rma package; then, the batch effect between the two datasets were corrected with the Limma and sva package. The metabolism-related genes in stroke were intersected form the up-regulated and down-regulated genes in both diseases. Principal component analysis (PCA) was performed to show the mixture of these datasets and eliminated batch effects.^42^ Differentially expressed genes (DEGs) between two groups were further identified with the Limma package. DEGs were considered at their log_2_FC > 1 and adjust *p* value <0.05.

#### GSVA analyses

Based on the “c2.cp.all.v7.0. symbols” gene set, we used the R package GSVA ^43^ to calculate the metabolic scores of the relevant pathways according to the gene expression matrix of each sample by the method of ssGSEA,^44^ and relevant pathways between the two clusters were listed in a heatmap, and adj.P.val<0.05 was considered statistically significant. Next, the relationship between the significant metabolic pathways and risk genes were screened with Pearson analysis.

#### Immune infiltration analysis

CIBERSORT is based on the principle of linear support vector regression to deconvolve the transcriptome expression matrix to estimate the composition and abundance of immune cells in mixed cell.^45^ We uploaded the gene expression matrix data to CIBERSORT, filtered samples with *p* > 0.05, and derived the immune cell infiltration matrix. Next, the relationship between the significant immune cells and risk genes were screened with Pearson analysis.

#### Weighted Co-Expression Network Analysis (WGCNA)

WGCNA analysis is a computational method to describe co-expressed genes between different groups.^45^^,^^46^ It can identify marker genes based on the non-orientation analysis between the gene set and phenotypes. Here, WGCNA was used to locate the gene modules related to the specific cluster in both ischemic stroke and metabolic syndrome.

#### Single cell sequencing data obtained and processing

The single-cell data were downloaded from NCBI website:GEO174574.^10^ Next, we carried out data quality control and captured cells with less than 10 percent mitochondrial genes, with a total number of genes ranged from 200 to 10000 and were expressed in at least three cells. The number of highly variable genes was set at 2000, which is consistent with previous studies.^38^^,^^47^^,^^48^ The six samples were integrated through SCT correction. Then, uMAP method were used to reduce the dimension of data. The single cell-RNA sequencing method was used to map the hub genes and locate their cell source.^49^ Cellchat R package (1.4.0) was applied to explore the cell-cell interaction between the cell clusters. We compared the glycolysis extent with addmodulescore method between each specific cell.

#### Pseudo-time analysis

The R package monocle v2.22.0 was applied for pseudo-time analysis to obtain the genes required for calculating differential gene expression from different pathological stages.^50^ After calculating the pseudo-time, the differential gene expression analysis was repeated to determine the genes that changed as a function of pseudo-time. The cell state containing the greatest number of S0-stage cells was considered as the root state. The threshold of the q value for multiple testing involved in the selection of DEGs was 0.01.

#### Receiver operating characteristic (ROC) and Precision-Recall curves (PRC) analysis

To identify the potential clinical significance of key genes, the diagnostic values of the model were evaluated by applying ‘pROC’, ‘randomForest’, ‘LASSO’ and ‘xgboost’ packages to construct the machine learning model. The ROC and PRA results were derived based on these analysis. A random forest^51^ is a machine learning algorithm that falls under the category of ensemble learning, which means it combines the predictions from multiple machine learning algorithms to make more accurate predictions than other individual models. XGBoost, which stands for eXtreme Gradient Boosting, is an advanced implementation of gradient boosting algorithm. XGBoost has been successfully applied to a wide range of supervised learning problems, often outperforming other methods in machine learning competitions and real-world tasks.^52^ Its success is due to both its performance in creating predictive and robust models and its speed in doing so. The Receiver Operating Characteristic (ROC) curve and the Precision-Recall (PR) curve are both graphical representations used to evaluate the performance of a classification model at various threshold settings. They are especially useful for binary classification problems where we deal with positive and negative classes. The ROC curve plots the True Positive Rate (TPR, also known as recall or sensitivity) against the False Positive Rate (FPR) at various threshold levels.^53^ ROC curves can present an overly optimistic view of the model’s performance if there is a large class imbalance. The PR curve plots Precision (positive predictive value) against Recall for various threshold levels.^54^ PR curves can provide a more informative picture of performance when there is a class imbalance, as they focus on the performance of the model in predicting the minority class. In practice, both ROC and PR curves are valuable tools, and it’s often beneficial to analyze both when evaluating classification models, especially in contexts where class imbalance might be influencing model performance.

#### MR study design

We applied the two-sample MR to evaluate the association between ischemic stroke and metabolic disorder. We extracted data from IEU website and set ebi-a-GCST005843 as exposure and finn-b-E4_METABOLIA as outcome data. We extracted single nucleotide polymorphisms (SNPs) for both diseases serving as instrumental variables (IVs) for each trait from previously published GWAS data.^55^^,^^56^^,^^57^ These SNPs were then clumped together to obtain independent genetic variants, ensuring a linkage disequilibrium (LD) r2 < 0.001 and a clumping distance of 1000 kb. For the selection of strong IVs, we considered a value of F-statistic larger than ten, which was deemed sufficient to predict the exposures of interest.^58^^,^^59^ When we do the bidirectional MR analysis, we set the finn-b-E4_METABOLIA as exposure data and ebi-a-GCST005843 as outcome. The study protocols were approved by the ethics committee of local hospital.

To ensure reliable harmonization of SNP-exposure and SNP-outcome, we followed previously described procedures.^60^ To address potential heterogeneity and horizontal pleiotropy across the causal estimates, we implemented five MR approaches: random-effect inverse-variance weighted (IVW), weighted median, MR-Egger, simple mode, and weighted mode. The IVW regression approach assumes that either all genetic variants are valid instruments or that there is no evidence of pleiotropy effect.^61^ Meanwhile, the weighted median analysis was utilized to assess robustness in consistent estimation if more than 50% of the instrumental variables are valid.^62^ To investigate potential horizontal pleiotropy, we conducted MR-Egger analysis, employing weighted linear regression between SNP-exposure and SNP-outcome, and detected its presence by examining the intercept of the MR-Egger coefficient.^63^ To evaluate heterogeneity, scatterplots were performed between causal estimates from multiple genetic variants.^64^ In sensitivity analyses, we compared the causal estimates from various MR methods, such as MR-Egger, penalized weighted median, simple mode, IVW, and weighted mode, to enhance the robustness of our findings. Forest plots were utilized to assess the causal effects of individual SNPs and to further compare them against the causal estimates derived from the IVW and MR-Egger approaches, which employed all enrolled SNPs. Furthermore, we examined possible directional pleiotropy by observing asymmetry in funnel plots to gauge the reliability of the current MR analyses.

### Quantification and statistical analysis

All data analyses were performed in R v4.1.3. Details of these bioinformatics analyses were described in corresponding subsections. The relationship between the significant immune cells and risk genes were screened for CIBERSORT with the *p* value <0.05. A *p* value <0.05 was defined as statistically significant.
