# Supplementary material for: Integrated single cell-RNA sequencing and Mendelian randomization for ischemic stroke and metabolic syndrome
Source: iScience. 2024 Jun 11;27(7):110240. doi: 10.1016/j.isci.2024.110240 (PMC11253530; doi:10.1016/j.isci.2024.110240)
Supplement: Document S1. Figures S1–S6 [file mmc1.pdf]

iScience, Volume *27*

Supplemental information

Integrated single cell-RNA sequencing and Mendelian randomization for ischemic stroke and metabolic syndrome

Jie Li, Sen Shen, Cong Yu, Shuchen Sun, and Ping Zheng

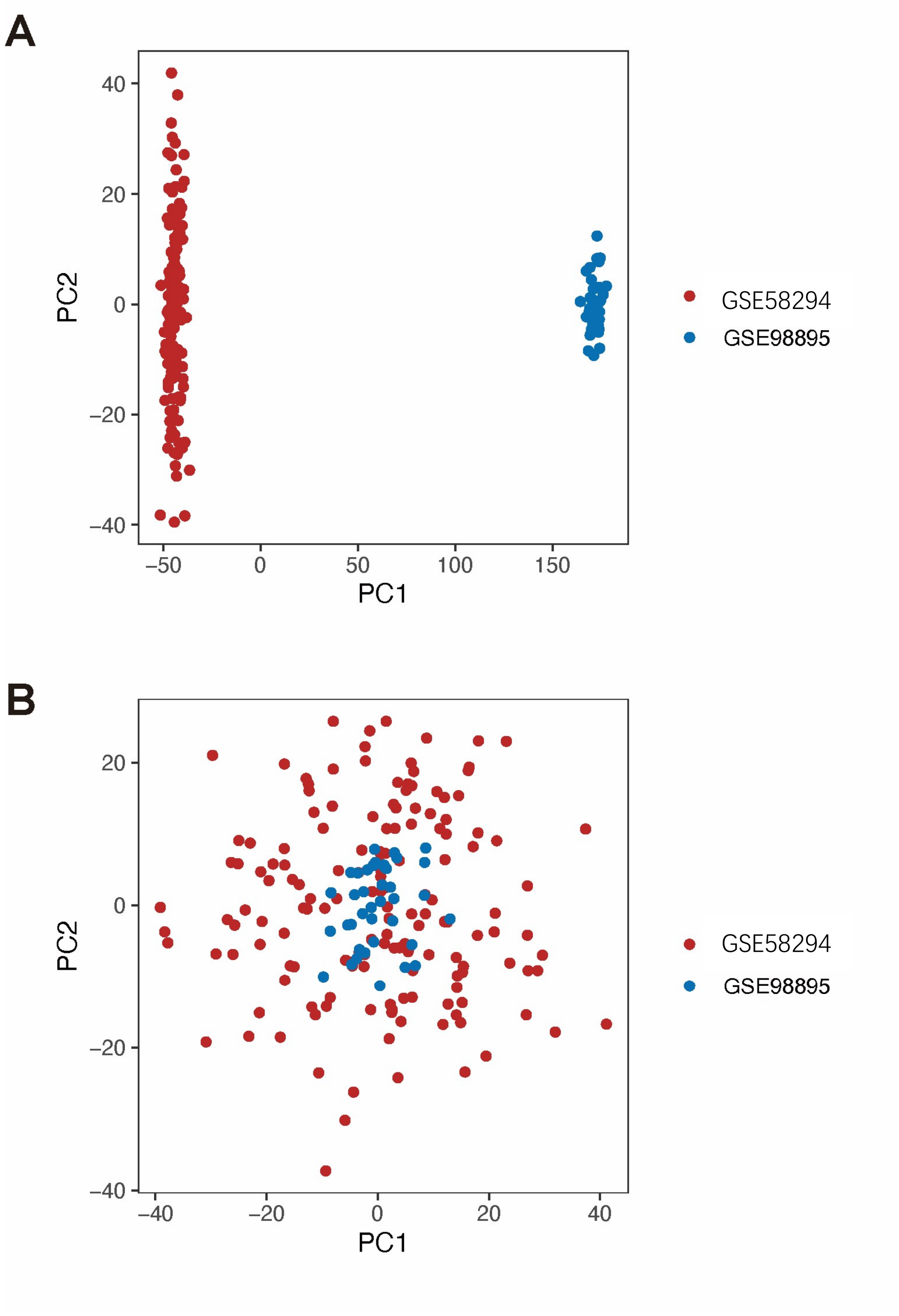
A
40
20
0
−20
−40
PC2
−50
0
50
100
150
PC1
GSE58294
GSE98895
B
20
0
−20
−40
PC2
−40
−20
0
20
40
PC1
GSE58294
GSE98895

Supp Fig 1.

A

```
elapsed = 0.02  Round = 1    eta = 0.0150222 max.depth = 6.0000    min_child_weight = 6.0000    subsample = 0.5734392   Value = 0.5000
elapsed = 0.035 Round = 2    eta = 0.02068883    max.depth = 8.0000    min_child_weight = 6.0000    subsample = 0.5212037   Value = 0.5000
elapsed = 0.025 Round = 3    eta = 0.02544456    max.depth = 7.0000    min_child_weight = 8.0000    subsample = 0.5298398   Value = 0.5000
elapsed = 0.026 Round = 4    eta = 0.01053561    max.depth = 8.0000    min_child_weight = 1.0000    subsample = 0.5948815   Value = 0.6875
elapsed = 0.019 Round = 5    eta = 0.02529552    max.depth = 6.0000    min_child_weight = 5.0000    subsample = 0.6555903   Value = 0.5000
elapsed = 0.019 Round = 6    eta = 0.04478763    max.depth = 7.0000    min_child_weight = 8.0000    subsample = 0.6986015   Value = 0.5000
elapsed = 0.018 Round = 7    eta = 0.02361396    max.depth = 7.0000    min_child_weight = 7.0000    subsample = 0.6220491   Value = 0.5000
elapsed = 0.019 Round = 8    eta = 0.0292832 max.depth = 8.0000    min_child_weight = 5.0000    subsample = 0.7738628   Value = 0.5000
elapsed = 0.049 Round = 9    eta = 0.03398263    max.depth = 7.0000    min_child_weight = 9.0000    subsample = 0.588081    Value = 0.5000
elapsed = 0.02  Round = 10   eta = 0.02974165    max.depth = 8.0000    min_child_weight = 5.0000    subsample = 0.6377197   Value = 0.5000
elapsed = 0.024 Round = 11   eta = 0.03479096    max.depth = 7.0000    min_child_weight = 1.0000    subsample = 0.5123926   Value = 0.6000
elapsed = 0.025 Round = 12   eta = 0.02480005    max.depth = 8.0000    min_child_weight = 1.0000    subsample = 0.5121009   Value = 0.7250
elapsed = 0.026 Round = 13   eta = 0.01881295    max.depth = 6.0000    min_child_weight = 1.0000    subsample = 0.7180016   Value = 0.6000
elapsed = 0.028 Round = 14   eta = 0.0500    max.depth = 8.0000    min_child_weight = 1.0000    subsample = 0.7875828   Value = 0.5000
elapsed = 0.02  Round = 15   eta = 0.02015886    max.depth = 8.0000    min_child_weight = 2.0000    subsample = 0.6942756   Value = 0.5375
elapsed = 0.021 Round = 16   eta = 0.02043802    max.depth = 8.0000    min_child_weight = 1.0000    subsample = 0.5353224   Value = 0.5750
elapsed = 0.022 Round = 17   eta = 0.02794739    max.depth = 6.0000    min_child_weight = 1.0000    subsample = 0.6873646   Value = 0.7375
elapsed = 0.024 Round = 18   eta = 0.0283771 max.depth = 8.0000    min_child_weight = 1.0000    subsample = 0.6678065   Value = 0.8000
elapsed = 0.025 Round = 19   eta = 0.02845636    max.depth = 8.0000    min_child_weight = 2.0000    subsample = 0.6471276   Value = 0.6125
elapsed = 0.029 Round = 20   eta = 0.02809338    max.depth = 8.0000    min_child_weight = 1.0000    subsample = 0.7988455   Value = 0.7250

 Best Parameters Found:
Round = 18    eta = 0.0283771 max.depth = 8.0000    min_child_weight = 1.0000    subsample = 0.6678065   Value = 0.8000
```

B

```
elapsed = 0.079 Round = 1    eta = 0.0150222 max.depth = 6.0000    min_child_weight = 6.0000    subsample = 0.5734392   Value = 0.5000
elapsed = 0.031 Round = 2    eta = 0.02068883    max.depth = 8.0000    min_child_weight = 6.0000    subsample = 0.5212037   Value = 0.5000
elapsed = 0.02  Round = 3    eta = 0.02544456    max.depth = 7.0000    min_child_weight = 8.0000    subsample = 0.5298398   Value = 0.5000
elapsed = 0.043 Round = 4    eta = 0.01053561    max.depth = 8.0000    min_child_weight = 1.0000    subsample = 0.5948815   Value = 0.8625
elapsed = 0.046 Round = 5    eta = 0.02529552    max.depth = 6.0000    min_child_weight = 5.0000    subsample = 0.6555903   Value = 0.5000
elapsed = 0.031 Round = 6    eta = 0.04478763    max.depth = 7.0000    min_child_weight = 8.0000    subsample = 0.6986015   Value = 0.5000
elapsed = 0.021 Round = 7    eta = 0.02361396    max.depth = 7.0000    min_child_weight = 7.0000    subsample = 0.6220491   Value = 0.5000
elapsed = 0.021 Round = 8    eta = 0.0292832 max.depth = 8.0000    min_child_weight = 5.0000    subsample = 0.7738628   Value = 0.5000
elapsed = 0.022 Round = 9    eta = 0.03398263    max.depth = 7.0000    min_child_weight = 9.0000    subsample = 0.588081    Value = 0.5000
elapsed = 0.042 Round = 10   eta = 0.02974165    max.depth = 8.0000    min_child_weight = 5.0000    subsample = 0.6377197   Value = 0.5000
elapsed = 0.027 Round = 11   eta = 0.02862926    max.depth = 8.0000    min_child_weight = 1.0000    subsample = 0.5236452   Value = 0.9250
elapsed = 0.057 Round = 12   eta = 0.0500    max.depth = 8.0000    min_child_weight = 1.0000    subsample = 0.6942061   Value = 0.9000
elapsed = 0.028 Round = 13   eta = 0.03571316    max.depth = 6.0000    min_child_weight = 1.0000    subsample = 0.7658222   Value = 0.9000
elapsed = 0.027 Round = 14   eta = 0.03533056    max.depth = 8.0000    min_child_weight = 1.0000    subsample = 0.6229139   Value = 0.9000
elapsed = 0.025 Round = 15   eta = 0.02145962    max.depth = 6.0000    min_child_weight = 1.0000    subsample = 0.7439509   Value = 0.8375
elapsed = 0.028 Round = 16   eta = 0.0500    max.depth = 8.0000    min_child_weight = 1.0000    subsample = 0.8000    Value = 0.8250
elapsed = 0.029 Round = 17   eta = 0.0500    max.depth = 6.0000    min_child_weight = 1.0000    subsample = 0.5000    Value = 0.9125
elapsed = 0.028 Round = 18   eta = 0.0500    max.depth = 8.0000    min_child_weight = 1.0000    subsample = 0.5000    Value = 0.8000
elapsed = 0.027 Round = 19   eta = 0.01209411    max.depth = 6.0000    min_child_weight = 1.0000    subsample = 0.5000    Value = 0.9375
elapsed = 0.029 Round = 20   eta = 0.0500    max.depth = 6.0000    min_child_weight = 1.0000    subsample = 0.6590579   Value = 0.8875

 Best Parameters Found:
Round = 19    eta = 0.01209411    max.depth = 6.0000    min_child_weight = 1.0000    subsample = 0.5000    Value = 0.9375
```

Supp Fig 2.

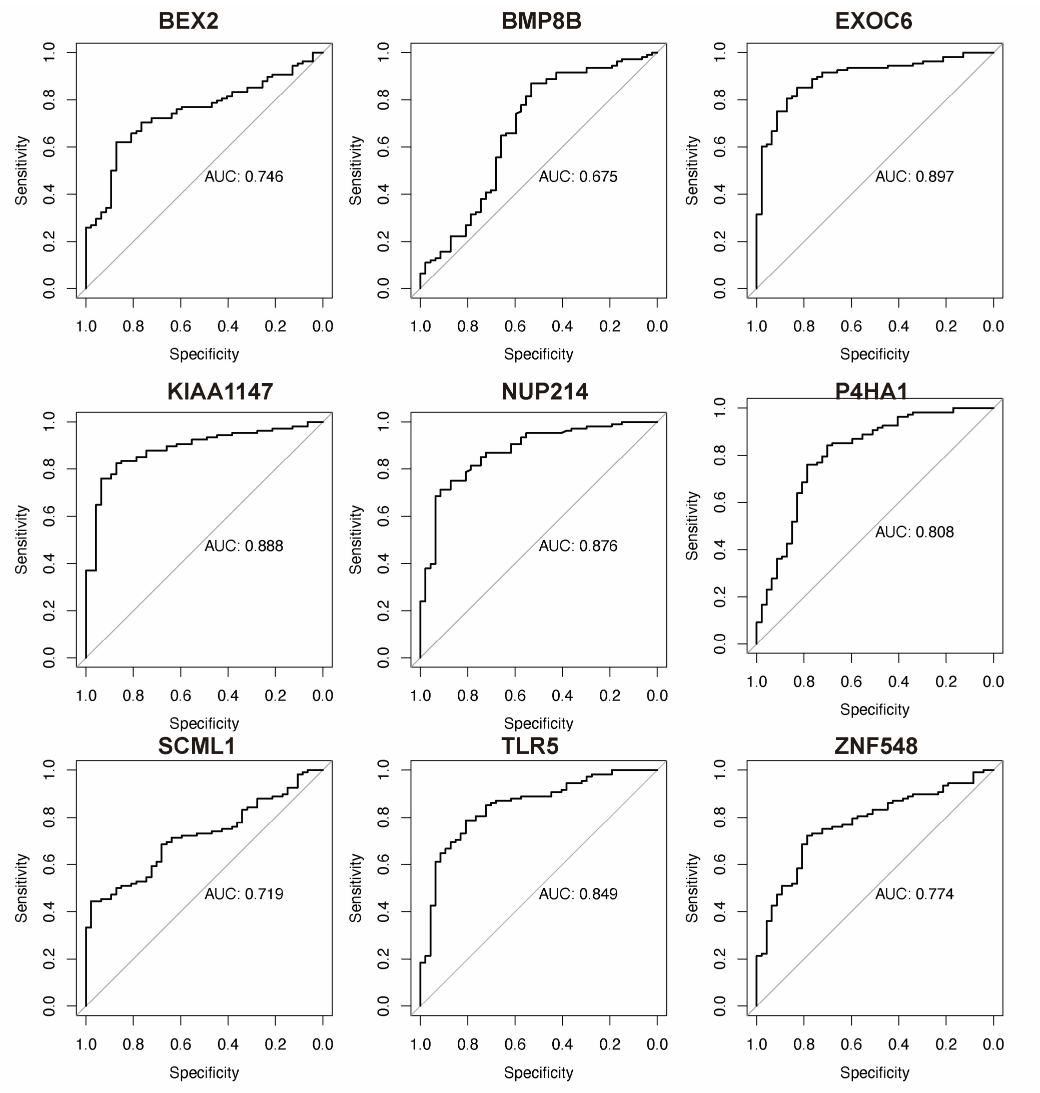
BEX2
AUC: 0.746
BMP8B
AUC: 0.675
EXOC6
AUC: 0.897
KIAA1147
AUC: 0.888
NUP214
AUC: 0.876
P4HA1
AUC: 0.808
SCML1
AUC: 0.719
TLR5
AUC: 0.849
ZNF548
AUC: 0.774
Sensitivity
Specificity

Supp Fig 3.

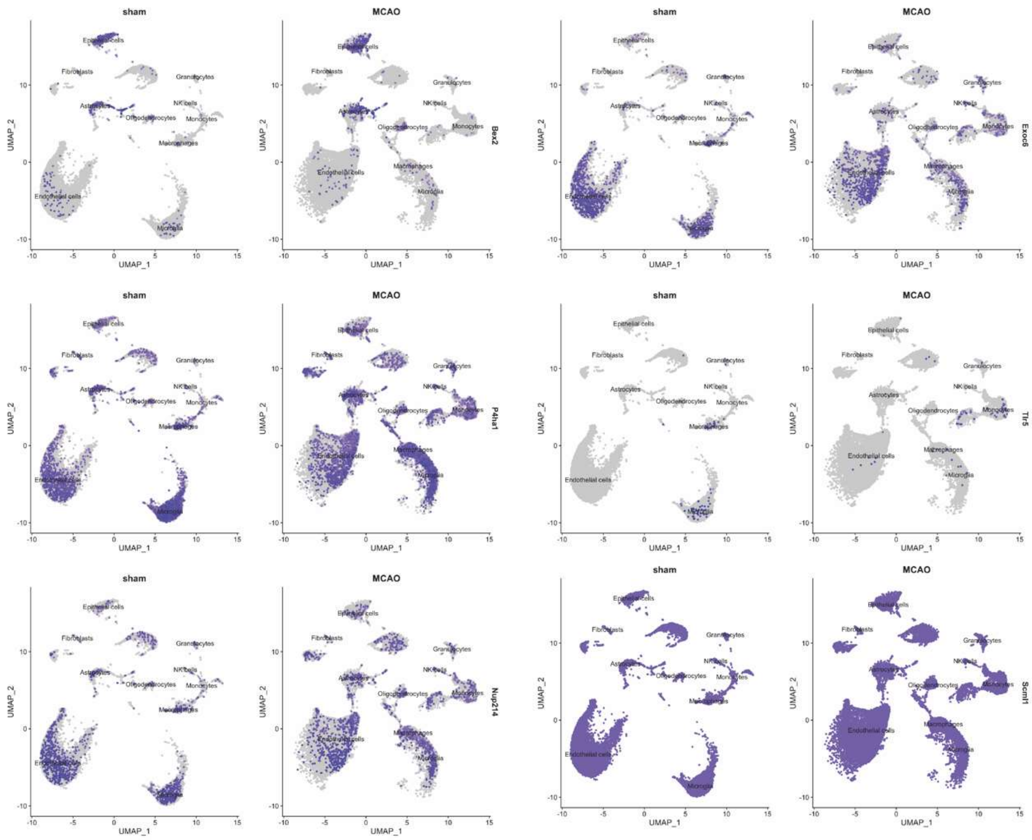

sham
MCAO
Bex2
Exoc6
P4ha1
Tlr5
Nup214
Scml1
Epithelial cells
Fibroblasts
Granulocytes
Astrocytes
NK cells
Oligodendrocytes
Monocytes
Macrophages
Endothelial cells
Microglia
UMAP_1
UMAP_2

Supp Fig 4.

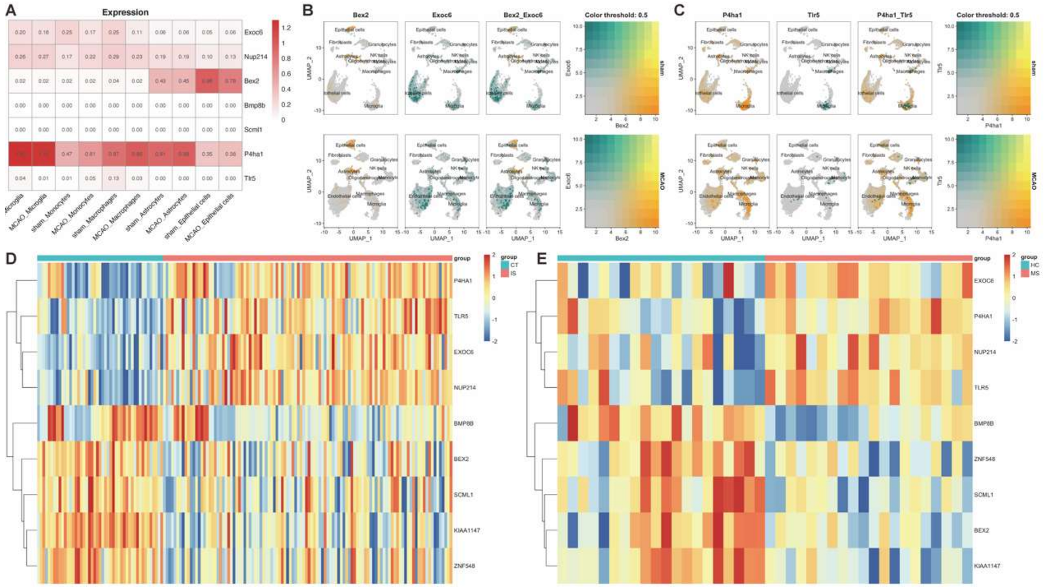

Supp Fig 5.

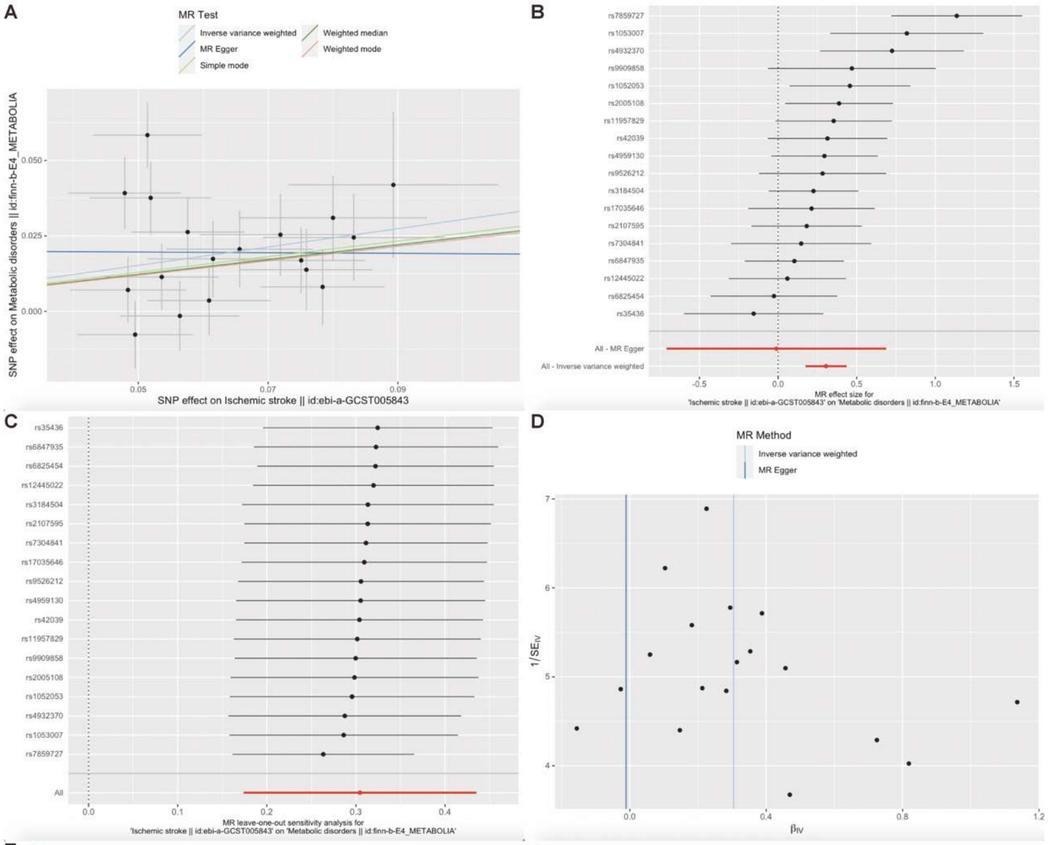

E

```
> OR
        id.exposure       id.outcome                                         outcome                             exposure
1 finn-b-E4_METABOLIA ebi-a-GCST005843 Ischemic stroke || id:ebi-a-GCST005843 Metabolic disorders || id:finn-b-E4_METABOLIA
2 finn-b-E4_METABOLIA ebi-a-GCST005843 Ischemic stroke || id:ebi-a-GCST005843 Metabolic disorders || id:finn-b-E4_METABOLIA
3 finn-b-E4_METABOLIA ebi-a-GCST005843 Ischemic stroke || id:ebi-a-GCST005843 Metabolic disorders || id:finn-b-E4_METABOLIA
4 finn-b-E4_METABOLIA ebi-a-GCST005843 Ischemic stroke || id:ebi-a-GCST005843 Metabolic disorders || id:finn-b-E4_METABOLIA
5 finn-b-E4_METABOLIA ebi-a-GCST005843 Ischemic stroke || id:ebi-a-GCST005843 Metabolic disorders || id:finn-b-E4_METABOLIA
                     method nsnp         b         se         pval       lo_ci     up_ci       or   or_lci95 or_uci95
1                  MR Egger   11 0.2295800 0.17848215 2.304442e-01 -0.12024502 0.5794050 1.258072 0.8867032 1.784976
2           Weighted median   11 0.2139799 0.05462885 8.966639e-05  0.10690739 0.3210525 1.238598 1.1128312 1.378578
3 Inverse variance weighted   11 0.1938545 0.06847502 4.639902e-03  0.05964349 0.3280656 1.213920 1.0614581 1.388280
4               Simple mode   11 0.2148435 0.11780689 9.818293e-02 -0.01605800 0.4457450 1.239668 0.9840702 1.561653
5             Weighted mode   11 0.2179712 0.08247052 2.460609e-02  0.05632902 0.3796135 1.243551 1.0579457 1.461719
```

Supp Fig 6.

Figure legends for supplementary files

Supp Figure 1. The PCA results of mixed datasets. (A) The batch effect of the two datasets. (B) Eliminated batch effect of the two datasets, related to Fig 1.

Supp Figure 2. The XGBoost optimization procedure in the external validated stroke and MS model, related to Fig 2. (A) The round 18 was selected for the XGBoost optimization in the external validated stroke model. (B) The round 19 was selected for the XGBoost optimization in the external validated MS model, related to Fig 2.

Supp Figure 3. The ROC of each hub gene in the stroke model, related to Fig 2.

Supp Figure 4. The six risk genes were mapped in the UMAP, related to Fig 4.

Supp Figure 5. The expression comparison of hub genes in single cell-RNA seq and bulk-seq dataset. (A) The expression comparison of hub genes at the single cell level. (B&C) The co-expressed genes in specific cell clusters. (D&E) The hub gene expression in stroke and MS model, related to Fig 4.

Supp Figure 6. Investigational of the reverse causal effects of ischemic stroke on the metabolic disorder using bidirectional MR methods. (A) The scatter plot of ischemic stroke on metabolic disorder. (B) The forest plot of ischemic stroke on metabolic disorder. (C) The leave-one-out method of ischemic stroke on metabolic disorder. (D) The funnel plot of ischemic stroke on metabolic disorder. (E) The bidirectional analysis of ischemic stroke on metabolic disorder, related to Fig 5.
